# Associated factors for exertional heat exhaustion-related symptoms among amateur golfers in Japan: A retrospective case-control study

**DOI:** 10.1080/23328940.2025.2508489

**Published:** 2025-06-03

**Authors:** Yosuke Nagashima, Teruhiko Hisaoka, Tomonori Sato, Yoshitomo Ehara, Akiko Horikawa, Akiyo Shiohara, Ayana Mitsume, Shigeru Mineo, Hiroaki Yoshida

**Affiliations:** a Department of Health Science, Musashigaoka Junior College, Yoshimi, Saitama, Japan; b Kanto Golf Association, Chuo-ku, Tokyo, Japan; c College of Sport and Wellness, Rikkyo University, Niiza, Saitama, Japan; d Department of Human Development and Sport Science, Tokyo International University, Kawagoe, Saitama, Japan; e Department of Nutrition and Health Sciences, Faculty of Food and Nutritional Sciences, Toyo University, Asaka, Saitama, Japan; f Nutraceuticals Science Laboratory, Advanced Research Institutes, Bourbon Corporation, Kashiwazaki, Niigata, Japan

**Keywords:** Thermoregulation, environmental heat stress, risk assessment, perceived health factors, golf and heat stress

## Abstract

The associated factors for exertional heat stroke among amateur golfers remain poorly understood. We conducted a case-control study to examine exertional heat exhaustion (EHE) – related symptoms among amateur golfers in Japan using a self-administered questionnaire. Retrospective case-control study design. A web-based questionnaire was administered from September to November 2024. Data were collected on basic attributes, lifestyle habits, perceived health factors, perceived playing conditions, changes in eating behavior, and EHE-related symptoms. We performed a case-control analysis using a multivariate conditional logistic regression model. The explanatory variables included lifestyle habits, health factors, playing conditions, and eating behavior; the objective variables were EHE-related symptoms. Our study included 194 participants with EHE-related symptoms and 252 control participants. The following factors were significantly associated with EHE symptoms: perceived dehydration (adjusted odds ratio [AOR], 4.65, 95% confidence intervals (CI) 3.08–7.03); sleep deprivation (AOR, 4.38, 95% CI 2.84–6.77); loss of appetite (AOR, 4.32, 95% CI 2.84–6.58); accumulated fatigue (AOR, 3.26 95% CI 1.17–1.31); mental stress (AOR, 2.71, 95% CI 1.78–4.12); average rounds of golf (AOR 1.88, 95% CI 1.14–3.13); increased sports drink consumption (AOR 2.07, 95% CI 1.20–3.56); increased consumption of salt tablets and candies (AOR 1.99, 95% CI 1.29–3.05); and increased dietary supplement intake (AOR, 1.88, 95% CI 1.14–3.11).These findings suggest that amateur golfers should assess their physical condition before play and adjust their schedules accordingly, particularly in hot weather, to minimize the risk of heat-related illnesses.

## Introduction

As global temperatures continue to rise due to climate change, the risk of heat-related injuries in sports and recreational activities has become increasingly concerning. Japan, in particular, experiences high temperatures and humidity levels during summer months, with these conditions intensifying year after year [1]. Hot and/or humid environments negatively impact exercise performance and increase the risk of heat stress-related symptoms and exertional heat illness (EHI) [2,3]. Consequently, engaging in outdoor sports during summer months poses substantial health risks.

EHI encompasses a spectrum of conditions ranging from muscle cramps and heat exhaustion (EHE) to exertional heat stroke (EHS) [4]. EHS is characterized by central nervous system dysfunction (e.g. collapse, convulsions, and coma), and even death in patients with hyperthermia [5]. EHS is responsible for approximately 2% of all deaths related to sport [6]. Furthermore, 323 sports-related heat stroke cases in Victoria, Australia, over an 11-year period, with golf being the most common sport, accounting for 43 cases (13.3%) of heat stroke-related hospitalizations [7]. According to reports in Japan, 21 out of 88 (23.4%) requests for ambulance services at golf courses in the Chubu region were for heat stroke [8]. Regarding EHI risk in golf, the International Golf Federation states: While playing or caddying in golf tournaments provides reduced exercise intensity compared to athletics, which decreases the risk of heat-related illnesses, exercise under extreme heat and/or humidity conditions can be expected to have negative health consequences for some individuals [9]. Based on this information, it is believed that playing golf under extreme heat and humidity conditions may increase the risk of heat stroke due to recent environmental changes [10,11].

Previous reports on the prevalence and/or incidence of EHI among athletes participating in summer sports are primarily based on medical records [7,12]. The actual incidence and prevalence rates of EHE among athletes are likely underreported, as mild cases that do not require hospitalization often go undocumented. Protective behaviors are commonly determined by subjective sensations related to physiological changes [13]. Pryor et al. reported that individual susceptibility to EHS depends on several factors, some of which are modifiable [14]. Early EHI detection and rapid cooling reduce EHS-associated morbidity and mortality [15]. Therefore, promptly identifying and reporting even mild initial symptoms of EHE are crucial to prevent EHS. A recent study by Yamashita et al. assessed EHE-related symptoms prevalence using a self-administered questionnaire among 2006 university students. The questions focused specifically on EHE symptoms, as EHE poses the primary risk of progressing to EHS. Revealing that 64.9% of male Japanese athletes had experienced EHE-related symptoms. Furthermore, the study identified that health factors significantly impact EHE-related symptoms [16].

However, to date, despite existing research on EHI in other endurance-based or outdoor sports, no studies have specifically examined the prevalence of EHE-related symptoms among golfers [17]. Golf is predominantly played by middle-aged and older adults between 40 and 70 years old, representing a different demographic compared with previous studies involving college athletes [18]. Age is a known risk factor for EHI [19]. Unlike high-intensity sports, golf involves prolonged exposure to heat, often for several hours, with limited opportunities for shade or active cooling. Typical golf attire may also contribute to reduced evaporative cooling, further increasing the risk of heat stress. Therefore, we believe that factors of EHE-related symptoms identified in other athletes may not be directly applicable to golfers. A better understanding of these associations could help equip golfers with the necessary knowledge to self-manage early symptoms of EHE, ultimately preventing progression to more dangerous conditions such as EHE and EHS. Thus, our study investigated the associations between health factors, lifestyle habits, eating behavior, playing conditions and EHE-related symptoms among amateur golfers in Japan through a self-administered questionnaire. This study is the first to systematically investigate health factors, lifestyle habits, dietary behaviors, and environmental factors associated with EHE-related symptoms among golfers. By identifying novel risk factors and influence of cognition, this study offers important practical implications for both individual golfers and tournament organizers, highlighting the need for improved education on early symptom detection and potential schedule adjustments during summer months.

## Methods

### Study design and population

A retrospective case-control study was conducted across 494 golf clubs affiliated with the Kanto Golf Federation and driving ranges affiliated with the Kanto Golf Driving Range Association. The Kanto region was selected as the target area because it has a temperate climate that is the same as most of Japan and it houses approximately one-quarter of Japan’s golf courses and represents a diverse population of golfers. This study adhered to the guidelines outlined in the Declaration of Helsinki, and all procedures were approved by the Ethics Committee of Musashigaoka Junior College, Japan (No. 24–2, July 12 2024) and the Kanto Golf Association Golf Promotion Committee Medical Department Meeting. The survey was administered to amateur golfers from September 20 to November 30 2024. To recruit participants, paper recruitment forms were distributed at competition venues of the competitions hosted by the Kanto Golf Club and Kanto Golf Association. Additionally, the Kanto Golf Driving Range Association was invited to collaborate, and recruitment forms were posted at driving ranges throughout the Kanto region. Golfers who agreed to participate accessed the web-based survey by scanning the QR code for the Google Form URL using their mobile phones. Informed consent was obtained through the Google Form platform prior to data collection. Golfers who played one or more rounds per month were included in the analysis. Those who did not consent to participate, had incomplete responses, or had bone or joint disorders (e.g. back, knee, or hip) that could limit their ability to play were excluded. Researchers reviewed all responses to ensure they were complete and natural. If anything was unclear, participants were encouraged to ask questions to the researchers.

### Questionnaire survey

In this study, we developed a questionnaire based on previous research [16,17]. target period is May to September 2024. The questionnaire survey assessed variables including general characteristics, frequency of EHE-related symptoms, perceived health factors, lifestyle habits (i.e. athletic situation and sleeping habit), perceived eating behaviors and perceived environmental factors [12]. The definition of EHE onset varies between Japan and Western countries. In Japan, the onset of EHE is defined as the period when the individual first notices symptoms [20,21], whereas in Western countries, it is defined as the point when the athlete can no longer continue exercising effectively [4,21,22]. The Japanese definition was used in this study as the participants were amateur golfers in Japan. Categories for each item were established based on previous studies [16,17,20]. The survey collected general demographic information including sex, age, body mass, underlying diseases, competition participation, and competitive level. Age data were collected using the following brackets: (1) ≤18 years, (2) 19–20 years, (3) 21–22 years, (4) 23–29 years, (5) 30–39 years, (6) 40–49 years, (7) 50–59 years, (8) 60–69 years, (9) 70–79 years, and (10) 80 years or older. The categories were predetermined according to previous research on age and physique [7,19]. Based on previous findings that exertional heat fatigue during golf predominantly affects individuals in age groups ≤ 35 and ≥65 years [7], we stratified participants’ ages into three categories for analysis: (1) ≤39 years, (2) 40–59 years, and (3) ≥60 years. Body mass data were initially collected across eight intervals: (1) ≤39 kg, (2) 40–49 kg, (3) 50–59 kg, (4) 60–69 kg, (5) 70–79 kg, (6) 80–89 kg, (7) 90–99 kg, and (8) ≥100 kg. To ensure sufficient sample size, these were subsequently consolidated into four categories: (1) <49 kg, (2) 50–69 kg, (3) 70–89 kg, and (4) ≥90 kg.

### EHE-related symptoms

EHE-related symptoms were assessed through the following questions: “During playing golf in the summer, have you ever felt symptoms such as dizziness, headache, nausea, or feeling sluggish (tired easily)?” (1, reported, 2, not reported) To address potential underestimation of EHE risks, we administered a questionnaire focused on EHE-related symptom. Participants were categorized into two groups based on their responses: those reporting symptoms were categorized as the “EHE-related symptom experienced participants group,” while those reporting no symptoms were categorized as the “control participants group.”

### Health factors

Seven items assessed perceived changes in physical condition during the summer season, focusing on: (1) loss of appetite, (2) sleep deprivation, (3) sickness, (4) hangover, (5) dehydration during golf, (6) accumulated fatigue, and (7) mental stress. Participants responded with two choices.

### Lifestyle habits

The questionnaire focused on lifestyle habits, including athletic situation (three questions), sleeping habits (two questions), and eating habits (two questions). Athletic situation assessment included rounds of golf, exercise status, and exercise duration: (1) Average monthly rounds of golf (1: ≤10 rounds, 2: ≥11 rounds); (2) Weekly exercise frequency (1: Less than once per week, 2: Once or more per week); (3) Exercise duration per session (1: <0.5 hours, 2: ≥0.5 hours). Sleep habits evaluated sleep duration and air conditioning usage: (1) Average sleep duration in the summer (1: <6 hours, 2: ≥6 hours); (2) Air conditioning use during bedtime (1: Used, 2: not used). Eating habits assessed breakfast consumption before golf and daily consumption of meals consisting of staple food, main dish, and side dish: (1) Daily consumption of meals consisting of staple food, main dish, and side dish (1: Two times or fewer, 2: Three times).; (2) Pre-golf breakfast consumption (1: Sometimes skipped, 2: Always consumed). Participants responded with two choices.

### Eating behaviors

Eight questions assessed perceived changes in eating behavior during the summer season, focusing on: (1) sports drinks, (2) gels, (3) dairy products, (4) fruits, (5) pickled plums, (6) ice-cream, (7) salt candy and tablets, and (8) dietary supplements. Participants responded to these questions using a three-point scale: 1 increased consumption, 2 usual consumption, and 3 decreased consumptions. According to previous research, increased consumption of supplement is risk factor, whereas increased consumption of sports drinks protective factor [23,24]. Therefore, we have provided three response options.

### Environmental factors

Six items evaluated golfers’ playing conditions: (1) high ambient temperature, (2) excessive humidity, (3) strong solar radiation, (4) lack of ambient wind, (5) discomfort in clothing, and (6) insufficient rest breaks. Participants responded with two choices.

### Statistical analyses

We generated descriptive statistics and conducted a case-control analysis using chi-square and Fisher’s exact tests. When expected cell counts were below five, the Fisher’s exact test was used instead of the chi-square test. Variables associated with increased EHE-related symptoms in the univariate analysis at a statistical significance level of *p* < 0.10 were then included in a multivariate logistic regression model. A threshold of *p* < 0.10 was chosen to avoid prematurely excluding potentially relevant variables, ensuring a more comprehensive multivariate model. Adjusted odds ratios (AOR) and 95% confidence intervals (CI) were then calculated. Based on prior studies, sex [19], age [19], body mass [19], underlying disease [19], and competition participation [20,25] were selected as adjustment variables. For categorical variables, “usual consumption” was used as the reference category in dietary behaviors, and the absence of the respective condition was the reference for health factors, lifestyle habits, eating behaviors and environmental factors. Logistic regression analyses were conducted using the “Fit Model” platform in JMP. p-values from multivariate logistic regression below 0.05 were considered statistically significant. All statistical analyses were performed using JMP version 14.3.0 (SAS Institute Inc., Cary, NC, USA).

## Results

### Participant demographics

Out of 454 golfers surveyed, 8 did not consent to participate and 446 provided valid responses, resulting in a final analysis with 98.2% response rate. Table 1 summarizes the demographic characteristics of the respondents (*n* = 446). Males comprised *n* = 301 (67.5%) of the sample, while females accounted for *n* = 143 (32.1%). The age distribution showed that the largest group was 50–59 years (*n* = 130, 29.1%), followed by 60–69 years (*n* = 105, 23.5%) and 40–49 years (*n* = 69, 15.5%). A majority of participants (*n* = 278, 62.3%) had competition experience. A total of *n* = 194 (43.5%) of the participants reported having experienced EHE-related symptoms, while *n* = 252 (56.5%) reported no prior EHE-related symptoms experiences.

**Table 1.** Basic attributes of participants.

Characteristic		Total (*n* = 446)	
Sex			
	Male	301	(67.5)
	Female	143	(32.1)
	No answer	2	(0.4)
Age, years			
	≤18	62	(13.9)
	19–22	20	(4.5)
	23–29	17	(3.8)
	30–39	27	(6.1)
	40–49	69	(15.5)
	50–59	130	(29.1)
	60–69	105	(23.5)
	70–79	16	(3.6)
Body mass, kg			
	≤39	2	(0.4)
	40–49	36	(8.1)
	50–59	102	(22.9)
	60–69	139	(31.2)
	70–79	105	(23.5)
	80–89	12	(2.7)
	90–99	46	(10.3)
	≥100	4	(0.9)
Underlying disease			
	Present	70	(15.7)
	Absent	376	(84.3)
Participation in competitions			
	Participated	278	(62.3)
	Did not participate	168	(37.7)
Competitive level (only among participants with competition experience, *n* = 273)*			
	National	80	(28.8)
	Region	97	(34.9)
	Others	102	(30.3)
Reported experience of EHE-related symptoms			
	Reported	194	(43.5)
	Not reported	252	(56.5)

Data are presented as counts (percentages).

EHE: Exertional heat exhaustion.

## Health factors

Perceived accumulated fatigue” was the most commonly reported symptom during summer (*n* = 365, 81.8%), followed by “mental stress” (*n* = 270, 60.5%), “sleep deprivation” (*n* = 264, 59.2%), and “loss of appetite” (*n* = 196, 44.0%,).

**Figure 1.**
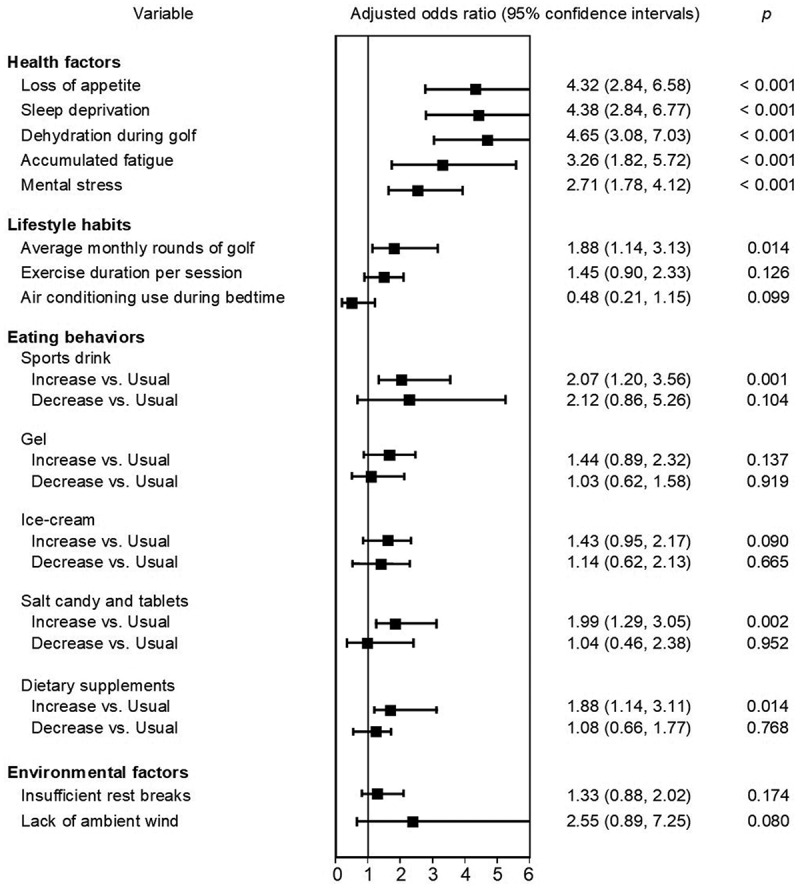
Factors associated with EHE-related symptoms. EHE: exertional heat exhaustion. The multivariate logistic regression analysis was used to calculate adjusted odds ratios and 95% confidence intervals. Odds ratios were adjusted for sex, age, body mass, underlying disease, and competition participation. The dependent variable was the EHE-related symptoms, and the independent variables were health factors, lifestyle habits, eating behaviors and environmental factors that were related to EHE-related symptoms. For categorical variables, “usual consumption” was used as the reference category in dietary behaviors, and the absence of the respective condition was the reference for health factors, lifestyle habits, eating behaviors and environmental factors.

In the univariate analysis (Table 2), the five items of dehydration, sleep deprivation, loss of appetite, accumulated fatigue, and mental stress were significantly associated with the risk of EHE-related symptoms. In multivariate analysis, the association between these health factors and EHE-related symptoms was also maintained (Figure 1); dehydration (AOR, 4.65, 95% CI 3.08–7.03, *p* < 0.001); sleep deprivation (AOR, 4.38, 95% CI 2.84–6.77, *p* < 0.001); loss of appetite (AOR, 4.32, 95% CI 2.84–6.58, *p* < 0.001); accumulated fatigue (AOR, 3.26, 95% CI 1.82–5.72, *p* < 0.001); and mental stress (AOR, 2.71, 95% CI 1.78–4.12, *p* < 0.001). Sickness and hangover were nonsignificant factors

**Table 2.** Association between experiencing EHE-related symptoms and basic attributes and health factors.

	Characteristic		Total (*n* = 446)		EHE-related symptom experienced participants (*n* = 194)		Control participants (*n* = 252)		*P*
	Basic attributes								
	Sex								
		Male	301	(67.5)	126	(65.0)	175	(69.4)	0.601^†^
		Female	143	(32.1)	67	(34.5)	76	(30.2)	
		No answer	2	(0.4)	1	(0.5)	1	(0.4)	
	Age, years								
		≤39	126	(28.3)	64	(33.0)	62	(24.6)	0.094
		40–59	199	(44.6)	85	(43.8)	114	(45.2)	
		≥60	121	(27.1)	45	(23.2)	76	(30.2)	
	Body mass, kg								
		≤49	38	(8.5)	11	(5.7)	27	(10.7)	0.064
		50–69	241	(54.0)	115	(59.3)	126	(50.0)	
		70–89	151	(33.9)	59	(30.4)	92	(36.5)	
		≥90	16	(3.6)	9	(4.6)	7	(2.8)	
	Underlying disease								
		Present	70	(15.7)	29	(15.0)	41	(16.3)	0.793
		Absent	376	(84.3)	165	(85.1)	211	(83.7)	
	Participation in competitions								
		Participated	278	(62.3)	128	(66.0)	150	(59.5)	0.169
		Did not participate	168	(37.7)	66	(34.0)	102	(40.5)	
	Competitive level (among competition participants, *n* = 273)								
		National	80	(28.8)	41	(32.0)	39	(26.0)	0.430
		Region	97	(34.9)	45	(35.2)	52	(34.7)	
		Others	102	(30.3)	42	(32.8)	59	(39.3)	
Health factors									
	Loss of appetite								
		Reported	196	(44.0)	123	(63.4)	73	(29.0)	<0.001
		Not reported	250	(56.0)	71	(36.6)	179	(71.0)	
	Sleep deprivation								
		Reported	264	(59.2)	149	(76.8)	115	(45.6)	<0.001
		Not reported	182	(40.8)	45	(23.2)	137	(54.4)	
	Sickness								
		Present	96	(21.5)	47	(24.2)	49	(19.4)	0.246
		Absent	350	(78.5)	147	(75.8)	203	(80.6)	
	Hangover								
		Present	82	(18.4)	34	(17.5)	48	(19.1)	0.723
		Absent	364	(81.6)	160	(82.5)	204	(81.0)	
	Dehydration during golf								
		Reported	200	(44.8)	127	(65.5)	73	(29.0)	<0.001
		Not reported	246	(55.2)	67	(34.5)	179	(71.0)	
	Accumulated fatigue								
		Reported	365	(81.8)	176	(90.7)	189	(75.0)	<0.001
		Not report	81	(18.2)	18	(9.28)	63	(25.0)	
	Mental stress								
		Reported	270	(60.5)	142	(73.2)	128	(50.8)	<0.001
		Not report	176	(39.5)	52	(26.8)	124	(49.2)	

Data are presented as counts (%). EHE: Exertional heat exhaustion.

Chi-squared test and Fisher’s exact test were used to compare differences in the experience of EHE-related symptoms for each variable. ^†^p-values was calculated using Fisher’s exact test.

## Lifestyle habits

The majority of participants reported sleeping at least 6 hours daily and used air conditioning while sleeping (*n* = 391, 87.7% and 417, 93.5%, respectively), and 83 participants (25.3%) played golf 11 or more times per month.

In the univariate analysis (Table 3). Average golf rounds, exercise duration, and use of air conditioning during bedtime were significantly positive associated with the risk of EHE-related symptoms. In multivariate analysis, the average golf rounds were risk factors for EHE-related symptoms (AOR, 1.88, 95% CI 1.14–3.13, *p* = 0.014). (Figure 1). Exercise duration and use of air conditioning were no longer a significant association in the multivariate model.

**Table 3.** Association between experiencing EHE-related symptoms and lifestyle habits and eating behaviors.

	Characteristic		Total (*n* = 446)		EHE-related symptom experienced participants (*n* = 194)		Control participants (*n* = 252)		*P*
Lifestyle habits									
	Average monthly rounds of golf								
		≤10 rounds	363	(74.7)	145	(74.7)	218	(86.5)	0.002
		≥11 rounds	83	(25.3)	49	(25.3)	34	(13.5)	
	Weekly exercise frequency								
		Less than once per week	110	(24.7)	42	(21.7)	68	(27.0)	0.223
		Once or more per week	336	(75.3)	152	(78.4)	184	(73.0)	
	Exercise duration per session, h								
		<0.5 hours	103	(23.1)	37	(19.1)	66	(26.2)	0.089
		≥0.5 hours	343	(76.9)	157	(80.9)	186	(73.8)	
	Average sleep duration in the summer								
		<6 hours	55	(12.3)	25	(12.9)	30	(11.9)	0.773
		≥6 hours	391	(87.7)	169	(87.1)	222	(88.1)	
	Air conditioning use during bedtime								
		Used	417	(93.5)	186	(95.9)	231	(91.7)	0.083
		Not used	29	(6.5)	8	(4.1)	21	(8.3)	
	Daily consumption of balanced meals								
		Two times or fewer	306	(68.6)	137	(70.6)	169	(67.1)	0.472
		Three times	140	(31.4)	57	(29.4)	83	(32.9)	
	Pre-golf breakfast consumption								
		Sometimes skipped	88	(19.7)	39	(20.1)	49	(19.4)	0.905
		Every day	358	(80.3)	155	(79.9)	203	(80.6)	
Eating behaviors									
	Sports drink								
		Increased consumption	339	(76.0)	158	(81.4)	181	(71.8)	0.023
		Usual consumption	78	(17.5)	23	(11.9)	55	(21.8)	
		Decreased consumption	29	(6.5)	13	(6.7)	16	(6.4)	
	Gels								
		Increased consumption	141	(31.6)	73	(37.6)	68	(27.0)	0.055
		Usual consumption	199	(44.6)	80	(41.2)	119	(47.2)	
		Decreased consumption	106	(23.8)	41	(21.1)	65	(25.8)	
	Dairy products								
		Increased consumption	25	(5.6)	12	(6.2)	13	(5.2)	0.308
		Usual consumption	303	(67.9)	125	(64.4)	178	(70.6)	
		Decreased consumption	118	(26.5)	57	(29.4)	61	(24.2)	
	Fruits								
		Increased consumption	63	(14.1)	30	(15.5)	33	(13.1)	0.704
		Usual consumption	303	(67.9)	128	(66.0)	175	(69.4)	
		Decreased consumption	80	(18.0)	36	(18.6)	44	(17.5)	
	Pickled plums								
		Increased consumption	114	(25.6)	58	(29.9)	56	(22.2)	0.157
		Usual consumption	251	(56.3)	105	(54.1)	146	(57.9)	
		Decreased consumption	81	(18.1)	31	(16.0)	50	(19.8)	
	Ice-cream								
		Increased consumption	190	(42.6)	94	(48.5)	96	(38.1)	0.087
		Usual consumption	197	(44.2)	76	(39.2)	121	(48.0)	
		Decreased consumption	59	(13.2)	24	(12.4)	35	(13.9)	
	Salt candy and tablets								
		Increased consumption	268	(60.1)	135	(69.6)	133	(52.8)	0.002
		Usual consumption	147	(33.0)	48	(24.7)	99	(39.3)	
		Decreased consumption	31	(6.9)	11	(5.7)	20	(7.9)	
	Dietary supplement								
		Increased consumption	90	(20.2)	49	(25.2)	41	(16.3)	0.064
		Usual consumption	264	(59.2)	107	(55.2)	157	(62.3)	
		Decreased consumption	92	(20.6)	38	(19.6)	54	(21.4)	

Data are presented as counts (%). EHE: Exertional heat exhaustion.

Chi-squared test was used to compare differences in the experience of EHE-related symptoms for each variable.

Exercise frequency, sleep duration, daily consumption of balanced meals and pre-golf breakfast consumption were not significantly associated with the risk of EHE-related symptoms.

### Eating behaviors

Of the participants, 76% (*n* = 339) reported increased consumption of sports drinks and 60.1% (*n* = 268) reported increased consumption of salt tablets and candies in the summer (Table 4).

**Table 4.** Association between experiencing EHE-related symptoms and environmental factors.

	Characteristic		Total (*n* = 446)		EHE-related symptom experienced participants (*n* = 194)		Control participants (*n* = 252)		*P*
Environmental factors									
	High ambient temperature								
		Reported	430	(96.4)	190	(97.9)	240	(95.2)	0.198
		Not report	16	(3.6)	4	(2.1)	12	(4.8)	
	Excessive humidity								
		Reported	425	(95.3)	187	(96.4)	238	(94.4)	0.356
		Not report	21	(4.7)	7	(3.6)	14	(5.6)	
	Strong solar radiation								
		Reported	431	(96.6)	190	(97.9)	241	(95.6)	0.289
		Not report	15	(3.4)	4	(2.1)	11	(4.4)	
	Lack of ambient wind								
		Reported	425	(95.3)	189	(97.4)	236	(93.7)	0.072
		Not report	21	(4.7)	5	(2.6)	16	(6.4)	
	Discomfort in clothing								
		Reported	311	(69.7)	140	(72.2)	171	(67.9)	0.350
		Not report	135	(30.3)	54	(27.8)	81	(32.1)	
	Insufficient rest breaks								
		Reported	163	(36.5)	81	(41.8)	82	(32.5)	0.048
		Not report	283	(63.5)	113	(58.3)	170	(67.5)	

Data are presented as counts (%). EHE: Exertional heat exhaustion.

Chi-squared test was used to compare differences in the experience of EHE-related symptoms for each variable.

In the univariate analysis (Table 3). There was no significant negative correlation between any of the eating behaviors and EHE-related symptoms. Unexpectedly, participants who reported the intake of sports drinks and, gels, ice-cream, salty candies, salt tablets, and dietary supplements were significantly positive associated with the risk of EHE-related symptoms. Additionally, in multivariate analysis showed that increased sports drink consumption (AOR, 2.07, 95% CI 1.20–3.56, *p* = 0.001); increased intake of salt tablets and candies (AOR, 1.99, 95% CI 1.29–3.05, *p* = 0.002); and dietary supplements (AOR, 1.88, 95% CI 1.14–3.11, *p* = 0.014) remained associated with the risk of EHE-related symptoms (Figure 1). Gels and ice-cream were no longer a significant association in the multivariate model. Young competitive golfers responded that they had had a high intake of these beverages and foods, it has been suggested that age and participation in competitions may influence this. Dairy products, fruits and pickled plums were nonsignificant factors.

## Environmental factors

Over 90% of participants experienced “high ambient temperature,” “excessive humidity,” “strong solar radiation,” and” lack of ambient wind,” and “insufficient rest breaks” was reported by 36.5% (*n* = 163) of participants).

In the univariate analysis (Table 4). The lack of ambient wind was significantly positive associated with the risk of EHE-related symptoms. However, lack of ambient wind was no longer a significant association in the multivariate model (Figure 1). The older participants were more likely they were to say that there was no wind, possibly indicating an effect of age.

In contrast, participants who reported insufficient rest breaks had a lower likelihood of experiencing EHE symptoms. This finding may suggest a reporting bias or an alternative mechanism requiring further investigation (Figure 1). High ambient temperature, excessive humidity, strong solar radiation and discomfort in clothing were nonsignificant factors.

## Discussion

Our case-control method revealed several risk factors associated with these EHE-related symptoms. Health factors demonstrated the strongest correlation, with the following independent risk factors identified in descending order of significance: dehydration, sleep deprivation, loss of appetite, accumulated fatigue, and mental stress. Additionally, lifestyle habits [average rounds of golf] and eating behaviors [consumption of sports drinks, salted candies, and supplements] were positively associated with EHE-related symptoms. These findings suggest that golfers should assess their physical condition before playing and consider adjusting their schedules accordingly, particularly in hot weather, to minimize the risk of heat-related illnesses.

This study used a questionnaire to assess the EHE-related symptoms among amateur Japanese golfers, revealing a rate of 43.5%. Previous studies using the same method to investigate the prevalence of EHE and EHI-related symptoms reported rates of 64.9% among male collegiate athletes [16], 44.7% among male university athletes [17], 31.9% among male Sports Science students, and 16.8% among male students from general departments [20]. Thus, the prevalence of EHE-related symptoms among amateur golfers in our study is within the range observed in collegiate athletes.

Regarding health factors, we found that “dehydration,” “sleep deprivation,” “loss of appetite,” “perceived accumulated fatigue,” and “perceived mental stress,” were positively associated with the occurrence of EHE-related symptoms. Our findings align with previous research and current statements from various institutions, indicating that poor physical condition (dehydration [21], sleep deprivation [21,26,27], and loss of appetite [19] may be associated with the risk of developing EHE-related symptoms. Our results indicate that this association between poor physical condition and EHE-related symptoms applies not only to athletes but also to recreational golfers.

Regarding lifestyle habits, only the average rounds showed a significant positive association with EHE-related symptoms. This aligns with previous position statements. The American College of Sports Medicine et al. (2007) reported that physical fatigue contributes to both the onset and progression of EHE-related symptoms [21]. An increase in golf rounds can lead to fatigue and exhaustion. Therefore, we hypothesize that increased rounds of golf may adversely affect mood state and contribute to accumulated fatigue, potentially increasing the risk of EHE-related symptoms. Given these findings, golfers should monitor their perceived fatigue levels and incorporate sufficient rest days to minimize EHE risk. Future research should explore the effectiveness of structured recovery protocols in amateur golf.

In our results, exercise time was not a factor associated with EHE-related symptoms. Previous studies have reported that moderate exercise or regular aerobic exercise in a cool environment has been shown to have the same effect as heat acclimation [13,28]. The results of this study do not agree with previous studies. However, it is not possible to conclude that exercise habits are unassociated with symptoms of EHE-related symptoms. Most of our respondents reported >0.5 h/week exercise duration; however, the exercise intensities were not assessed. Therefore, further research is warranted to clarify whether exercise duration with intensity contributes to the onset of EHE-related symptoms.

Therefore, further research is needed to clarify whether exercise intensity and duration are linked to the onset of EHE-related symptoms. Unlike previous studies, we found no significant relationship between air conditioning use during sleep [16,17,27] or sleep duration [16,17]. This discrepancy may be attributed to the fact that 93.5% of our study participants used air conditioning, with very few participants not using it during sleep, potentially limiting our ability to detect any significant associations.

Pre-golf breakfast consumption was not associated with whether athletes experienced EHE-related symptoms. Our findings contradict the Ministry of Environmental guidelines that recommend having breakfast to prevent EHI [29]. Although speculative, this can be explained by the fact that golf courses are typically located in suburban areas, requiring players to travel long distances early in the morning. Players often opt for a light meal or skip breakfast while traveling, choosing instead to snack during play. Therefore, in this study, skipping breakfast or meals consisting of staple foods, main dishes, and side dishes was not identified as a risk factor for EHE-related symptoms. However, it is not possible to draw any conclusions based on our results alone. Thus, further exploration of this finding is warranted, perhaps by analyzing the timing of breakfast or the type of food consumed.

Regarding eating behaviors, although increased consumption of sports drinks, salt tablets, and supplements was associated with higher EHE risk, this result likely reflects a compensatory response to early symptoms rather than a direct cause. This finding aligns with research showing that supplements can promote EHI [23], but contrasts with studies indicating that sports drinks and salt candies/tablets help prevent EHI [19,21,26,30]. Such relationships cannot be clarified in this study. Further research is needed to determine which specific supplements affect EHE-related symptoms.

Regarding environmental factors, no significant association was found for any of the items in the multivariate model. According to a review by Westland et al., climate was reported to be a major risk factor for EHI-related symptoms [4,19,21,26]. Additionally, in a previous study on college athletes, high temperatures, lack of wind, and short rest periods were positively associated with EHE-related symptoms. Our results were contradictory to previous research. Over 90% of participants experienced “high ambient temperature,” “excessive humidity,” “strong solar radiation,” and “lack of ambient wind.” The association between environmental factors and EHE-related symptoms may have been attenuated by the relatively extended time period covered by this study. Thus, research covering a shorter period of time is warranted.

In the univariate analysis, the association between insufficient rest breaks and being less likely to experience EHI-related symptoms, younger females golfers reported having insufficient rest breaks. Thus, sex and age may have influenced higher EHI-related symptoms. According to previous studies, sex and age were reported to be a major risk factor for EHI-related symptoms [31]. Thus, in the multivariate model adjusting for sex and age, the association was thought to have disappeared. Additionally, men and female have been reported to perceive different sensations [32,33]. This finding may suggest an alternative mechanism requiring further investigation.

This study demonstrated that various risk factors (health factors, lifestyle habits, eating habits) are associated with the incidence of EHE-related symptoms in amateur golfers. Our findings align with Westwood et al. (2021), who reported that health-related factors are associated with EHI [19]. Golfers who met our screening criteria (i.e. dehydration, sleep deprivation, loss of appetite) during pre-participation assessment may help prevent the onset of EHE. Any suspicious symptoms should be considered a sign of EHE, particularly when occurring during summer months [22]. Therefore, knowledge of EHE-related symptoms and self-diagnostic capabilities are crucial; golfers need proper education to accurately recognize the various symptoms indicating EHE. Furthermore, our findings suggest that individuals involved in golf competitions should adjust their hectic schedules to prevent overfatigue during summer months.

This study has several limitations. First, its retrospective case-control design may have introduced recall bias. Therefore, future prospective studies should employ a cohort study design and electronic daily logs to track exposure to lifestyle and health factors. Second, there was sampling bias, with disproportionate numbers across participant groups, although confounding factors were addressed through multivariate analysis. Golfers were recruited using distributed forms and QR codes at competition venues and driving ranges. This recruitment method may introduce self-selection bias, as individuals with prior EHE experiences or greater health awareness might be more likely to participate. Furthermore, the study focused exclusively on amateur golfers in Japan, wherein it was possible to compare the international golf population in different climates. However, this limited the generalizability of our findings to other populations, such as professional golfers, or individuals participating in other sports. Future studies should investigate golfers and athletes in various climates to identify the risk factors for EHE-related symptoms in broader populations. Third, we assessed EHE-related symptoms rather than EHE itself; therefore, the results may include symptoms that are not related to EHE, such as fever, upper respiratory tract viral infections, and physical discomfort associated with menstrual cycles. Fourth, the definition of EHE slightly differs between Japan and Western countries. Given that this study used the Japanese definition [21,26], our findings may not be applicable to athletes in Western countries. The Japanese definition of EHE onset, which differs from the Western definition, may have led to an underestimation of EHE symptom prevalence. Fifth, survey results were based on subjective sensations/perceptions (i.e. dehydration and sleep deprivation), which may be inconsistent with objective indices. To minimize these limitations, future research should incorporate objective measures for a more comprehensive assessment [34]. Some of these objective measures include wearable sensors to track deep body temperature and physical activity [35] as well as biochemical hydration markers for urine tests to assess dehydration. Sixth, age and body mass were treated as categorical rather than continuous variables, potentially affecting the observed model. This study did not collect information on exposure factors or the timing and frequency of heat stroke-related symptoms. Future research should gather and analyze such data.

## Conclusion

The strongest factors associated with the risk of developing EHE-related symptoms among amateur golfers were perceived dehydration, sleep deprivation, and loss of appetite, followed by perceived accumulated fatigue, perceived mental stress, and average rounds of golf played. Although increased consumption of sports drinks, salted candies, and supplements was associated with EHE-related symptoms, this likely reflects a compensatory response to early signs of heat stress rather than a direct causal link. Golfers should assess their physical condition before play and consider adjusting their schedules accordingly, particularly in hot weather, to minimize the risk of heat-related illnesses. Future efforts may be needed to educate golfers on early detection and accurate reporting of EHE-related symptoms. Additionally, tournament organizers should explore alternative scheduling strategies, such as early morning or evening sessions, to minimize heat exposure and reduce accumulated fatigue.

## Practical implications

- **Pay attention to how you feel**: Golfers should listen to their bodies, especially during hot weather. Feeling unusually tired, dehydrated, experiencing a loss of appetite, or mentally stressed could be warning signs of heat illness.
- **Don’t play through warning signs**: If golfers experience any of the warning signs mentioned above during practice/training or a round of golf, it is best to reduce your exercise level or adjust your schedule accordingly is necessary.
- **Frequent play might increase risk**: Playing many rounds of golf in a short period, especially in the summer, could increase your risk of heat illness due to accumulated fatigue. Consider spacing out your games. Consider that tournament organizers should consider explore alternative scheduling strategies, such as early morning or evening sessions.
- **Be mindful of sports drinks and salt supplements**: While staying hydrated is important, relying too heavily on sports drinks, salt tablets, or other supplements might not always prevent heat illness and could even be a sign that you’re already at risk.
- **Stay informed**: Golfers should be aware of the symptoms of heat exhaustion, such as dizziness, headache, nausea, and feeling sluggish. Recognizing these symptoms early can help prevent more serious problems.

## Data Availability

The datasets generated and/or analyzed during this study are not publicly available because our ethical approval did not include the use of these data by other researchers. The materials pertinent to this study are available from the corresponding author upon reasonable request.

## Abbreviations

AOR: Adjusted odds ratio
CI: Confidence intervals
EHE: Exertional heat exhaustion
EHI: Exertional heat illness
EHS: Exertional heat stroke

## Appendix (survey form)

Please select one item from the list below:

**1**. **Basic attributes**

**1–1. What is your sex?**

- Male
- Female Please number box
- I have no answer

**1–2. What is your age? (as of September 20, 2024)**

- ≤18 years
- 19–22 years
- 23–29 years
- 30–39 years
- 40–49 years
- 50–59 years
- 60–69 years
- 70–79 years Please number box
- 80 years or older

**1–3. What is your body mass?**

- ≤39 kg
- 40–49 kg
- 50–59 kg
- 60–69 kg
- 70–79 kg
- 80–89 kg
- 90–99 kg Please number box
- ≥100 kg

**1–4. Do you have an underlying disease*?**

*Underlying diseases (diabetes, hypertension, cardiac disease, kidney disease, extensive skin disease, thyroid disease)

- Present Please number box
- Absent

**1–5. Do you participate in competitive golf?**

- Participated Please number box
- Did not participate

**1–6. Your competitive standard***

**[Only answer if you selected (1) for questions 1–5]**

*Highest competitive result in 2024

- National
- Region Please number box
- Others

**2**. **Physical problems while playing golf**

**2–1. During playing golf in the summer** (**May to September), have you ever felt symptoms such as dizziness, headache, nausea, or malaise (tired easily)?**

- Reported Please number box
- Not reported

**3**. **Lifestyle habits**

**3–1. How many rounds of golf on average are played in a month during the summer (May to September) of 2024?**

- ≤10 rounds Please number box
- ≥11 rounds

**3–2. How often did you do exercise in the summer of 2024 (May to September)*?**

- Less than once per week Please number box
- Once or more per week

**3–3. How long was your exercise duration per session in the summer of 2024 (May to September)?**

- <0.5 hours Please number box
- ≥0.5 hours

**3–4. On average, how much time did you spend sleeping during the summer of 2024 (May to September)?**

- <6 hours Please number box
- ≥6 hours

**3–5. Did you use the air conditioner while you slept?**

- Used Please number box
- Not used

**3–6. How often did you eat breakfast before playing golf in the summer of 2024 (May through September)?**

- Sometimes skipped Please number box
- Always consumed

**3–7. How many “staple”, “main”, and “side” meals did you eat per day in your normal life during the summer of 2024 (May through September)?**

- Two times or fewer Please number box
- Three times

**4**. **Health factors**

How often did you experience the following symptoms while playing golf during the summer of 2024 (May through September)?

**4–1. Loss of appetite**

- Reported Please number box
- Not reported

**4–2. Sleep deprivation**

- Reported Please number box
- Not reported

**4–3. Sickness (i.e. a cold)**

- Present Please number box
- Absent

**4–4. Hangover**

- Present Please number box
- Absent

**4–5. Dehydration during golf (i.e. thirst)**

- Reported Please number box
- Not reported

**4–6. Accumulated fatigue**

- Reported Please number box
- Not reported

**4–7. Mental stress**

- Reported Please number box
- Not reported

**5**. **Eating behaviors**

How did you consume the following foods and beverages while playing golf in the summer of 2024 (May through September) compared to other periods (October 2023 through April 2024)?

**5–1. Sports drink**

- Increased consumption Please number box
- Usual consumption
- Decreased consumption

**5–2. Gels**

- Increased consumption Please number box
- Usual consumption
- Decreased consumption

**5–3. Milk and dairy products**

- Increased consumption Please number box
- Usual consumption
- Decreased consumption

**5–4. Fruits**

- Increased consumption Please number box
- Usual consumptionDecreased consumption

(3)

**5–5. Pickled plums**

- Increased consumption Please number box
- Usual consumption
- Decreased consumption

**5–6. Ices**

- Increased consumption Please number box
- Usual consumption
- Decreased consumption

**5–7. Salt candy and salt tablets**

- Increased consumption Please number box
- Usual consumption
- Decreased consumption

**5–8. Dietary supplement**

- Increased consumption Please number box
- Usual consumption
- Decreased consumption

Thank you for taking the time to fill in this for
